# Intranasal Immunisation with Recombinant *Toxoplasma gondii* Actin Partly Protects Mice against Toxoplasmosis

**DOI:** 10.1371/journal.pone.0082765

**Published:** 2013-12-27

**Authors:** Li-Tian Yin, Hai-Xia Hao, Hai-Long Wang, Jian-Hong Zhang, Xiao-Li Meng, Guo-Rong Yin

**Affiliations:** 1 Department of physiology, Key Laboratory of Cellular Physiology Co-constructed by Province and Ministry of Education, Shanxi Medical University, Taiyuan, Shanxi, PR China; 2 Research Institute of Medical Parasitology, Shanxi Medical University, Taiyuan, Shanxi, PR China; 3 General Hospital of the Datong Coal Mine Co. Ltd., Datong, Shanxi, PR China; University of Melbourne, Australia

## Abstract

*Toxoplasma gondii* is a ubiquitous protozoan intracellular parasite, the causative agent of toxoplasmosis, and a worldwide zoonosis for which an effective vaccine is needed. Actin is a highly conserved microfilament protein that plays an important role in the invasion of host cells by *T. gondii*. This study investigated the immune responses elicited by BALB/c mice after nasal immunisation with a recombinant *T. gondii* actin (rTgACT) and the subsequent protection against chronic and lethal *T. gondii* infections. We evaluated the systemic response by proliferation, cytokine and antibody measurements, and we assessed the mucosal response by examining the levels of TgACT-specific secretory IgA (SIgA) in nasal, vaginal and intestinal washes. Parasite load was assessed in the liver and brain, and the survival of mice challenged with a virulent strain was determined. The results showed that the mice immunised with rTgACT developed high levels of specific anti-rTgACT IgG titres and a mixed IgG1/IgG2a response with a predominance of IgG2a. The systemic immune response was associated with increased production of Th1 (IFN-γ and IL-2), Th2 (IL-4) and Treg (IL-10) cytokines, indicating that not only Th1-type response was induced, but also Th2- and Treg-types responses were induced, and the splenocyte stimulation index (SI) was increased in the mice immunised with rTgACT. Nasal immunisation with rTgACT led to strong mucosal immune responses, as seen by the increased secretion of SIgA in nasal, vaginal and intestinal washes. The vaccinated mice displayed significant protection against lethal infection with the virulent RH strain (survival increased by 50%), while the mice chronically infected with RH exhibited lower liver and brain parasite loads (60.05% and 49.75%, respectively) than the controls. Our data demonstrate, for the first time, that actin triggers a strong systemic and mucosal response against *T. gondii*. Therefore, actin may be a promising vaccine candidate against toxoplasmosis.

## Introduction

*Toxoplasma gondii*, the causative agent of toxoplasmosis, is a widespread opportunistic pathogen capable of invading virtually all nucleated cells of warm-blooded animals. It is estimated that human infection rates are extremely high, with at least one-third of people being infected worldwide [1], [2]. Although toxoplasmosis is generally asymptomatic in healthy individuals, it may provoke severe problems in immunodeficient individuals, such as AIDS patients, whereby *T. gondii* infection can cause encephalitis, pneumonia and disseminated infections [3]. When pregnant women are infected with *T. gondii* for the first time during pregnancy, toxoplasmosis may be transmitted transplacentally, possibly leading to neonatal malformations, neurological damage, blindness or foetal death [4], [5]. Meanwhile, *T. gondii* infection in farm animals has considerable economic importance, as it causes abortion, stillbirth and neonatal loss, especially in sheep [6]. Consumption of raw or undercooked meat containing *T. gondii* tissue cysts from infected intermediate hosts is the main source of parasite transmission to humans and continues to be a public health concern [7]. Currently, *T. gondii* control depends primarily on chemotherapy, but the available drugs have many side effects along with problems of reactivation. The poor results of the available treatment options have led to pleas for developing an effective vaccine [8]. Although one commercial vaccine based on the live attenuated S48 strain has been used for livestock [9], the live vaccine is poorly characterised at the genetic level and carries an inherent risk of reverting to virulence [10]. The development of safe and effective vaccines is the best strategy for the prevention of toxoplasmosis [11], [12].

The mucosa is the largest immune organ in the body, and almost all infectious diseases are initiated at a mucosal surface [13]. Injected vaccines can induce strong systemic immune responses but are not very efficient at inducing immune responses at mucosal sites, the primary route by which *Toxoplasma* infects its host. Mucosal delivery has considerable potential for improving the effectiveness of vaccination against local pathogens by increasing immunity at the sites of entry [14]. The intranasal route requires fewer antigens than the oral route because there is less proteolytic activity in the nasal cavity [15]. This route effectively promotes the systemic and mucosal immune responses to a given antigen [16]. Nasal vaccine innovation comes with both opportunities and challenges [17]. A number of studies have been carried out to investigate the potential of utilising the intranasal route for the induction of protective immune responses, and they have provided encouraging results [15], [18]–[20].

*Toxoplasma* lacks locomotion organelles, but it displays highly dynamic gliding movements over the substratum without changing its cell shape [21]–[23]. *T. gondii* invades cells through an active process that is dependent on actin-myosin interactions [24], [25]. Actin is a highly conserved microfilament protein that plays an important role in the invasion of host cells by *T. gondii* [26], [27].

The recombinant *T. gondii* actin (rTgACT) protein was produced in *Escherichia coli* and showed specific antigenicity in our study [28]. To our knowledge, this is the first study to utilise the *T. gondii* actin gene or protein as an antigen. It can be presumed that when the TgACT protein is used as an antigen, TgACT-specific antibodies will likely bind the tachyzoite actin antigens, impairing the ability of the tachyzoites to invade host cells. Therefore, the efficacy of *T. gondii* tachyzoite invasion may be reduced or even blocked.

To assess whether mice immunised with rTgACT induced immune protection against *T. gondii* infection, we investigated the systemic and mucosal immune responses elicited by BALB/c mice after nasal immunisation with rTgACT and protection against chronic and lethal *T. gondii* infections.

## Materials and Methods

### Toxoplasma gondii strain

Tachyzoites of the virulent *T. gondii* RH strain were used to challenge immunised mice, and preparations of *T. gondii* antigens were provided by Peking University Health Science Center (Beijing, China). The parasites were maintained and collected from the peritoneal cavity of infected specific-pathogen-free (SPF) BALB/c mice as previously described [20].

### Expression and purification of the recombinant protein

The rTgACT was expressed in *E. coli* strain BL21 (DE3) and purified from inclusion bodies by affinity chromatography using nickel-nitrilotriacetic acid (Ni-NTA) agarose (Qiagen, Germany) as described previously [28]. Briefly, the open reading frame of TgACT gene was amplified with a pair of specific primers which was designed according to the coding sequence of TgACT gene (NCBI Reference Sequence: XM_002369622.1), the product of RT-PCR was cloned into the prokaryotic expression pET-30a(+) vector. The recombinants pET-30a(+)-TgACT plasmid was transferred into *E. coli* DH5α and the positive clones were selected through the colony-PCR and confirmed by the double restrict enzyme digestion and sequencing. The successful pET-30a(+)-TgACT construct was transformed into *E. coli* BL21 (DE3) and induced with IPTG to express. The expressed proteins were analysed by SDS-PAGE, and the antigenicity of rTgACT was analysed with rabbit antiserum of *T. gondii*. The endotoxin in the recombinant protein was removed by using ToxinEraserTM Endotoxin Removal Kit, and the endotoxin level was measured with the Chromogenic End-point Endotoxin Assay Kit (Chinese Horseshoe Crab Reagent Manufactory, Xiamen, China). Less than 0.1 EU/ml was detected in the final protein preparations. Before inoculation into mice or stimulation *in vitro*, rTgACT was dialysed against PBS, filtered through a 0.2 µm-pore membrane and stored at −70°C. The purified recombinant protein was quantified by the Bradford method.

### Mice, ethics statement and immunisation

Female BALB/c mice were purchased from the Institute of Laboratory Animals, Chinese Academy of Medical Science (Beijing, China). All the mice were maintained under specific-pathogen-free conditions and provided with rodent feed and water *ad libitum*. Prior to experiments, the mice were acclimatised for one week. The animal protocols were approved by the Laboratory Animal Use and Care Committee of Shanxi Medical University (Permit Number: SXMU-2011-16) and the Ethics Committee on Animal Research of the Shanxi Medical University (Protocol #: 20110320-1). For immunisation experiments, 50 female BALB/c mice aged 6 weeks were randomly divided into 5 groups. The mice were immunised nasally with 20 µl of phosphate buffer saline (PBS) containing 10, 20, 30 or 40 µg of rTgACT and instilled slowly into both the nostrils (10 µl per nostril) with a micropipette, while the control mice were given PBS. All the animals were vaccinated three times on days 0, 14, and 21.

### Blood samples and mucosal washes collections

Prior to collecting the samples, the mice were deprived of food for 8 h to deplete the intestinal contents. Blood samples were collected from the retroorbital plexus of mice anesthetised with sodium pentobarbital 15 days after the last immunisation. Serum was obtained after centrifugation and stored at −70°C for further analysis. Nasal, vaginal and intestinal washes were collected using PBS. For the collection of nasal washes, the trachea was exposed surgically, a micropipette was then passed through the larynx and nasal cavity, sterile PBS was injected into the tracheal opening near the throat, liquid was passed through the throat and the nasal cavity and the flow from the two nostrils was collected into a test tube. A total of 0.5 ml of sterile PBS was flushed five times (0.1 ml per time) per mouse. For the collection of vaginal washes, a micropipette tip was inserted into the opening of the vulva of a euthanised mouse, 0.6 ml of sterile PBS was flushed gently in and out of the vaginal tract and all the fluid was collected by a micropipette. Each mouse was flushed six times (0.1 ml per time). A total of 0.1 ml of PBS was pulled back into the micropipette and reinfused into the vagina for a total of three cycles before the final withdrawal. For the collection of intestinal washes, the small intestine was removed from the proximal end of the duodenum and the distal portion of the ileum and was transversally cut into approximately 3-cm pieces. These pieces were flushed three times with a total of 3 ml of sterile PBS using a micropipette. The mucosal washes were collected in 1.5 ml or 5 ml, respectively, in an Eppendorf tube and were immediately centrifuged at 12,000× *g* for 30 min at 4°C. All the samples were stored at −20°C until assayed for antibody titres.

### Lymphocyte proliferation assay

The spleens were aseptically removed from the mice (six per group) 2 weeks after the last immunisation and were pressed through stainless steel meshes in Hank's balanced salt solution (HBSS, Sigma). Single-cell preparations were pelleted and resuspended in erythrocyte lysis buffer (0.15 M NH_4_Cl, 1.0 M KHCO_3_, 0.1 mM EDTA, pH 7.2). After centrifugation at 110×*g* for 10 min at 4°C, the pelleted cells were washed three times in PBS and resuspended in 2 ml of PBS. The cells were underlayed with two millilitres of lymphocyte separating solution (Shanghai Heng Chemical Reagents, Ltd, China), and the tube was centrifuged at 450×*g* for 10 min at RT. The lymphocyte layer was carefully transferred into a fresh 5-ml polypropylene centrifuge tube, the pelleted cells were washed two times in PBS and centrifuged at 110×*g* for 10 min at 4°C, the supernatant was then aspirated and discarded, and the remaining cell pellet was diluted to 1 ml with RPMI-1640 complete medium. The lymphocytes were counted on a haemocytometer.

The cells were then plated in triplicate in flat-bottom 96-well microtitre plates at a density of 5×10^5^ cells per well and were cultured in the presence of rTgACT (10 µg/ml), Concanavalin A (Con A; 5 µg/ml; Sigma; positive control) or medium alone (negative control) at 37°C in a 5% CO_2_ incubator. The plates were incubated for 72 h and pulsed with 10 µl of CCK-8 reagent (Dojindo Laboratories; Kumamoto, Japan) per well for the final 4 h. The absorbance was measured in the triplicate cultures at 450 nm to quantitatively evaluate cell viability. The stimulation index (SI) was calculated as the ratio of the average OD_450_ value of wells containing antigen-stimulated cells to the average OD_450_ value of wells containing cells with medium. All the assays were performed in triplicate.

### Cytokine assays

Cytokines were measured as previously described [29], [30]. Spleen cells were obtained as described above and cultured by triplicate in flat-bottom 24-well microtitre plates. Supernatants from the cultured splenocytes (1.5×10^6^) were collected after 24, 72 or 96 h of stimulation with rTgACT (10 µg/ml) and assayed for interleukin-2 (IL-2) and IL-4 at 24 h, for IL-10 at 72 h, and for interferon-gamma (IFN-γ) at 96 h. IL-2, IL-4, IL-10 and IFN-γ concentrations were determined using a commercial ELISA Kit (PeproTech, USA) according to the manufacturer's instructions. All the assays were performed in triplicate. The sensitivity limits of detection of IL-2, IL-4, IL-10 and IFN-γ were 16, 16, 47 and 23 pg/ml, respectively.

### Specific IgG and SIgA detection

The serum samples and mucosal washes were tested for the presence of specific IgG and secretory IgA (SIgA) by ELISA. Ninety-six-well polystyrene plates (Corning) were coated with 7.5 µg/ml rTgACT (100 µl/well) in PBS overnight at 4°C. The plates were washed with PBS containing 0.05% Tween20 (PBST), blocked for 1 hour at 37°C in PBS containing 5% FCS, and then washed with PBS. Thereafter, the serum samples (1∶200) and mucosal washes were incubated in different wells (100 µl/well) for 1 h at 37°C. After washing, the wells were incubated with 100 µl of goat anti-mouse HRP antibody (AbD Serotec; diluted 1∶2500 in PBS) for serum specific IgG or goat anti-mouse HRP antibody (Sigma; diluted 1∶1000 in PBS) for 1 h at 37°C. The plates were washed extensively and incubated with 100 µl of substrate solution for 30 min at 37°C. The optical density was measured at 492 nm (OD_492_) with a microplate reader (Bio-Tek) followed by 50 µl 2 N H_2_SO_4_ to stop the enzyme reaction. All the samples were run in triplicate.

### Challenge infection

A total of 40 female BALB/c mice aged 6 weeks were randomly divided into two groups (20 mice per group) and vaccinated intranasally with 20 µl PBS or 30 µg rTgACT at days 0, 14, and 21. This rTgACT immunisation dose and the immune programme were based on the results of the above-mentioned immunisation experiment. On day 15 after the last immunisation, 8 and 12 mice in each group were challenged orally (with a feeding needle) with 1×10^4^ tachyzoites of the RH strain for chronic assay and 4×10^4^ tachyzoites for acute infection. On the 30th day after challenge, the chronically infected mice were anesthetised with sodium pentobarbital, and the numbers of tachyzoites in the murine livers and brains were measured to evaluate the immunoprotective effect of vaccination. For the acute challenge, the survival of the challenged mice was monitored and recorded twice daily until 30 days after the parasite challenge.

### Tachyzoite loads of livers and brains

On day 30 after the parasite challenge, the liver central lobule and whole brain of each chronically infected mouse were removed and weighed to evaluate the tachyzoite load as our previously described [31]. Tissue homogenates were obtained by crushing the liver or brain through a 41.6 µm stainless steel mesh sieve, adding PBS (total 2 ml) drop by drop. The tissue homogenates were centrifuged at 1000×*g* for 15 min at RT, the precipitates were resuspended in 2 ml of PBS and were then centrifuged at 1000×*g* for 15 min at RT. Two millilitres of PBS containing 2.5% trypsinase were added, and the tissues were digested in a water bath at 37°C for 30 min. The suspensions were underlayed with two millilitres of room-temperature (RT) lymphocyte separating solution (Shanghai Heng Chemical Reagents, Ltd, China), the tube was centrifuged at 1400×*g* for 30 min at RT, the precipitate were resuspended in 1 ml of PBS, and the mean number of tachyzoites in the brain and liver were determined by counting four samples of 25 µl aliquots under an optical microscope. The tachyzoite load was estimated on the basis of the average quantity of tachyzoites per gram brain or liver tissue.

In order to verify the accuracy of evaluating the tachyzoite load above method, the distributions of tachyzoite in the liver tissues were measured using a sensitive real-time quantitative PCR (qRT-PCR) method as previously described [32]. Briefly, genomic DNA from the purified parasites and collected liver samples (100 mg) were extracted using a UniversalGen DNA Kit (CWBIO, China) according to the manufacturer's instructions. The forward and reverse sequences of SAG1 gene were 5′-CTGATGTCGTTCTTGCGATGTGGC-3′ and 5′-GTGAAGTGGTTCTCCGTCGGTGT-3′, respectively. The SYBR Green fluorescence was detected using the Applied Biosystems® Real-Time PCR Instruments. Each reaction mixture contained 12.5 µl of UltraSYBR Mixture (CWBIO), 0.4 µl of each primer (20 µM), 1.0 µl of DNA template and 10.7 µl of sterile distilled water. Sterile water was used as negative control, and a DNA extracted from 500 tachyzoites of the RH strain per ml was used as positive control. All reactions were performed in triplicate and PCR conditions were as follows: 95°C for 1 min, 40 cycles at 95°C for 5 s, and 60°C for 15 s and 72°C for 10 s.

The number of parasites in the samples was calculated from the qPCR threshold cycle (Ct) value according to a standard curve (linear curve slope: -3.3756, Y intercept: 45.8402, R^2^: 0.9973) obtained with DNA samples from a range of serial 10-fold dilutions (5×10^0^–5×10^7^/ml) of RH strain tachyzoites.

### Statistical analysis

Data, including antibodies, cytokine and lymphocyte proliferation were analysed by one-way analysis of variance (ANOVA). The survival time and tachyzoite loads were analysed by the Kaplan-Meier using SPSS software. Levels of significance of the differences between groups were determined by the Student's unpaired *t* test. Two-sided P values<0.05 were considered to indicate statistical significance. Tests of normality for the data within each group were analysed by the Shopiro-Vilk, *P* values were >0.10.

## Results

### Systemic immune response induced by rTgACT vaccination

To assess the systemic immune response in the immunised mice, the levels of antigen-specific IgG, IgG1 and IgG2a antibodies in the sera and cytokines from the spleen cell supernatants were evaluated by ELISA.

**Figure 1:**
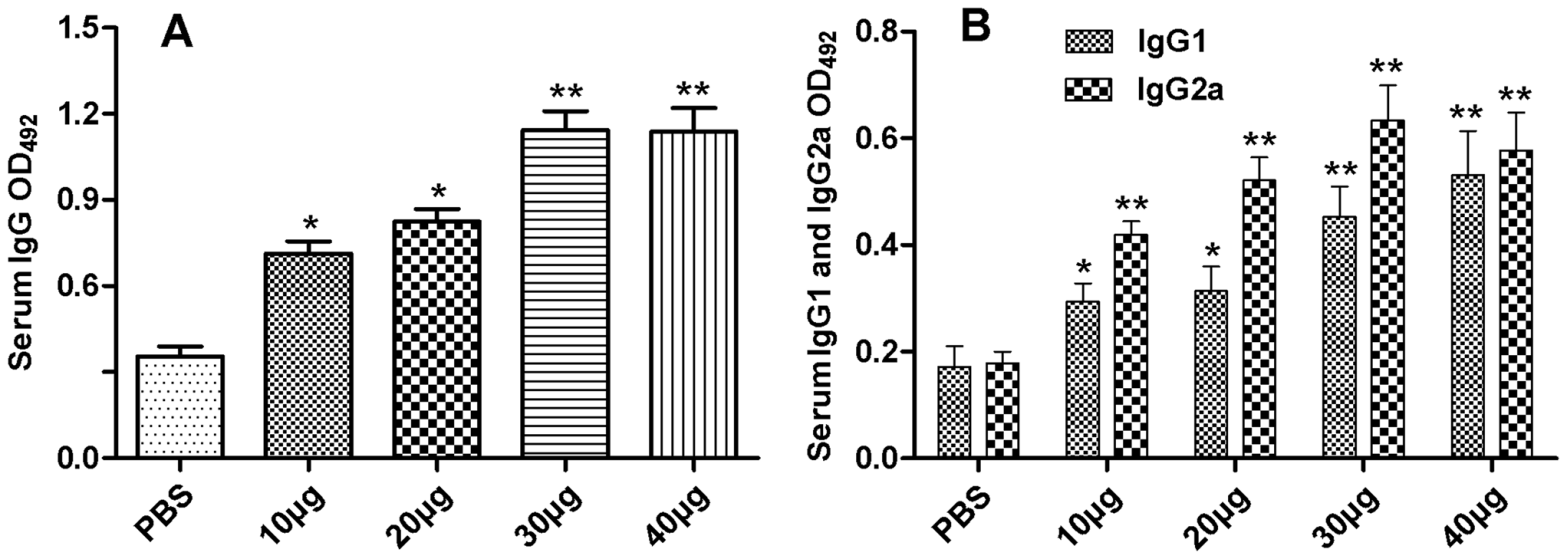
Nasal immunisation induces rTgACT-specific IgG responses in sera. Titres of both specific total IgG and IgG isotype antibodies in the sera of BALB/c mice were determined by ELISA with rTgACT as the bound target two weeks after the last immunisation. (A) Specific total IgG and (B) IgG1 and IgG2a titres in the sera of mice vaccinated with rTgACT. The results are expressed as the means of the OD_492_ ± SD (n = 10) and are representative of three experiments. *P<0.05, ** *P*<0.01 (vaccinated vs. PBS group).

The total IgG antibody response of the mice immunised with rTgACT was significantly higher compared to the control group (*P*<0.01) (Fig. 1A). The results showed that 30 µg and 40 µg of rTgACT could elicit the maximum IgG antibody responses compared to the 10 µg and 20 µg rTgACT groups (*P*<0.05), but no significant differences were observed in the IgG responses between higher and lower dose groups (*P*>0.05). A mixed IgG1/IgG2a response with predominant IgG2a production was detected in the sera of mice immunised with rTgACT (Fig. 1B). In general, the level of TgACT-specific IgG2a was greater than that of IgG1. These results indicated a shift toward the Th1 type response. Moreover, the mice immunised with all the doses of rTgACT elicited higher levels of IgG2a compared to the controls (*P*<0.01). The 30 µg group was the highest but was not significantly different than the 20 µg and 40 µg groups (*P*>0.05). These findings confirmed the results obtained with the anti-*T. gondii* IgG subclass, indicating that the cellular immune response was oriented to a Th1 profile in the immunised mice.

Two weeks after the last immunisation, spleen cells were prepared to assess the systemic proliferative responses to rTgACT. The splenocytes stimulation index (SI) from the mice immunised with 20, 30 and 40 µg rTgACT were higher than those immunised with 10 µg rTgACT and PBS (*P<*0.01) (Table 1), and the SI in the 10 µg group was also significantly higher than that of the PBS groups (*P<*0.05). However, there were no significant differences between the SIs of the high dose groups (30 and 40 µg) (*P*>0.05). In addition, splenocytes from all the experimental and control groups proliferated to comparable levels in response to ConA (data not shown). These results demonstrate that nasal administration of rTgACT triggered a systemic cell-mediated immune response.

**Table 1:** Lymphocyte proliferation and cytokine production by splenocytes stimulated with rTgACT.

Groups#	Lymphocyte SI	Cytokine production (pg/ml)##			
		IFN-γ	IL-2	IL-4	IL-10
PBS	0.29±0.08	94.24±10.79	112.57±13.37	118.83±7.63	225.04±10.22
10 µg rTgACT	0.58±0.10^a^	152.80±8.50^a^	225.65±25.68^a^	178.90±14.44	265.60±16.55
20 µg rTgACT	1.16±0.16^b^	172.76±14.98^b^	269.58±11.95^b^	201.99±13.12	305.62±16.41
30 µg rTgACT	1.77±0.13^b^	185.54±11.82^b^	300.81±23.59^b^	246.45±9.06^b^	348.65±10.52^a^
40 µg rTgACT	1.72±0.13^b^	173.43±15.26^b^	297.27±32.62^b^	207.70±12.68^a^	324.64±11.26^a^

^#^ *n* = 6 per group.

## Splenocytes from mice were harvested 2 weeks after the last immunisation. The results are presented as the arithmetic means ± standard errors of three replicate experiments. Values for IFN-γ are for 96 h, values for IL-2 and IL-4 are for 24 h, and values for IL-10 are for 72 h.

a : *P<*0.05 vs. control;

b : *P<*0.01 vs. control.

Cell-mediated immunity was evaluated by measuring cytokine levels (IFN-γ, IL-2, IL-4 and IL-10) in the supernatants of rTgACT-stimulated spleen cell cultures. Significantly higher levels of IFN-γ and IL-2 were observed in the spleen cell cultures from the mice immunised with 20, 30 and 40 µg rTgACT compared to the mice immunised with PBS (*P<*0.01) (Table 1). The 30 µg group was slightly higher among the three dose groups, but it was not significantly different. Meanwhile, the levels of IFN-γ and IL-2 in the 20 µg group were also higher than that of the PBS group (*P<*0.05). Similarly, the secretions of IL-4 and IL-10 in the splenocyte supernatants from immunised mice were also increased. The IL-4 levels in the 20, 30 and 40 µg groups were higher than those of the other groups, and the 30 µg group was the highest among the three dose groups (*P<*0.01). The levels of IL-10 showed a slight but significant increase in the splenocytes from the mice immunised with 30 µg and 40 µg rTgACT compared to those from the mice immunised with low dose rTgACT or PBS (*P<*0.05). These findings demonstrate that nasal administration of rTgACT induced a strong systemic immune response *in vivo* and *in vitro*, and a dose-dependent pattern was confirmed.

### Mucosal immune responses induced by rTgACT vaccination

**Figure 2:**
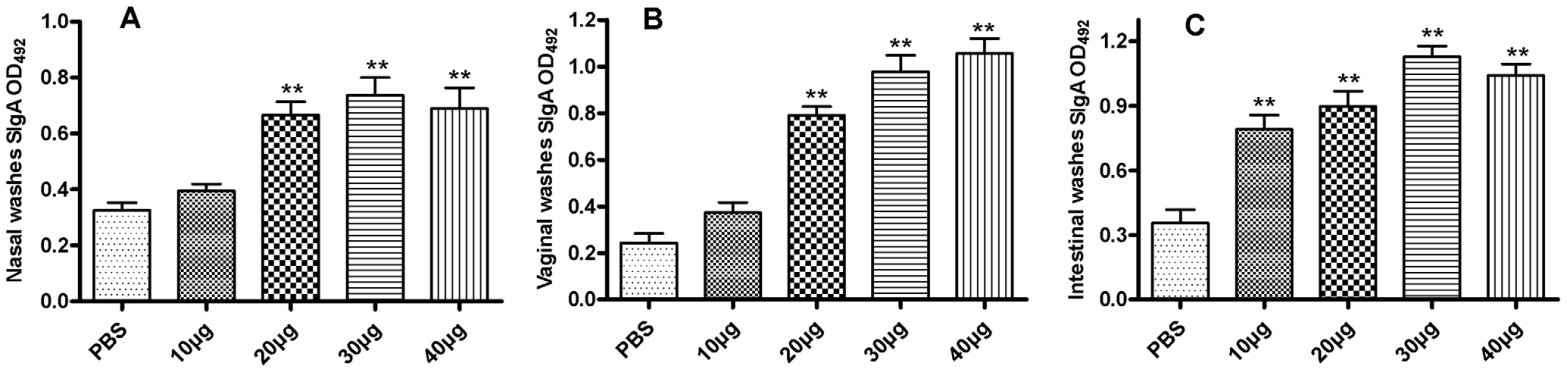
Nasal immunisation induces rTgACT-specific SIgA responses in mucosal washes. The SIgA antibody titres in mucosal washes from the mice were tested by ELISA two weeks after the last immunisation. High-level SIgA in (A) nasal washes, (B) vaginal washes and (C) intestinal washes were induced in mice nasally immunised with rTgACT compared to those vaccinated with PBS. **: Compared to PBS group, *P<*0.01. There were 6 mice per group, and the values are expressed as the mean ± SD. Significant differences were seen at *P*<0.01 (**) compared to mice immunised with PBS.

To investigate whether the mice immunised with rTgACT induced mucosal immune responses, the levels of specific SIgA in the mucosal washes were tested by ELISA two weeks after the last immunisation. The rTgACT-specific SIgA antibody titres in the mucosal washes were increased following nasal immunisation (Fig. 2). The SIgA antibody titres in the nasal, vaginal and intestinal washes were higher in the mice nasally immunised with rTgACT compared with the PBS control. The SIgA antibody titres in the nasal and vaginal washes in the 20, 30 and 40 µg rTgACT groups were significantly higher than those of the other groups (*P<*0.01), and the SIgA antibody titres in the intestinal washes in all the immunisation groups were significantly higher than that of the control group (*P<*0.01). Therefore, strong mucosal immune responses were induced by nasal immunisation with rTgACT at nasal, vaginal and intestinal mucosal sites.

### Protection against oral challenge

**Figure 3:**
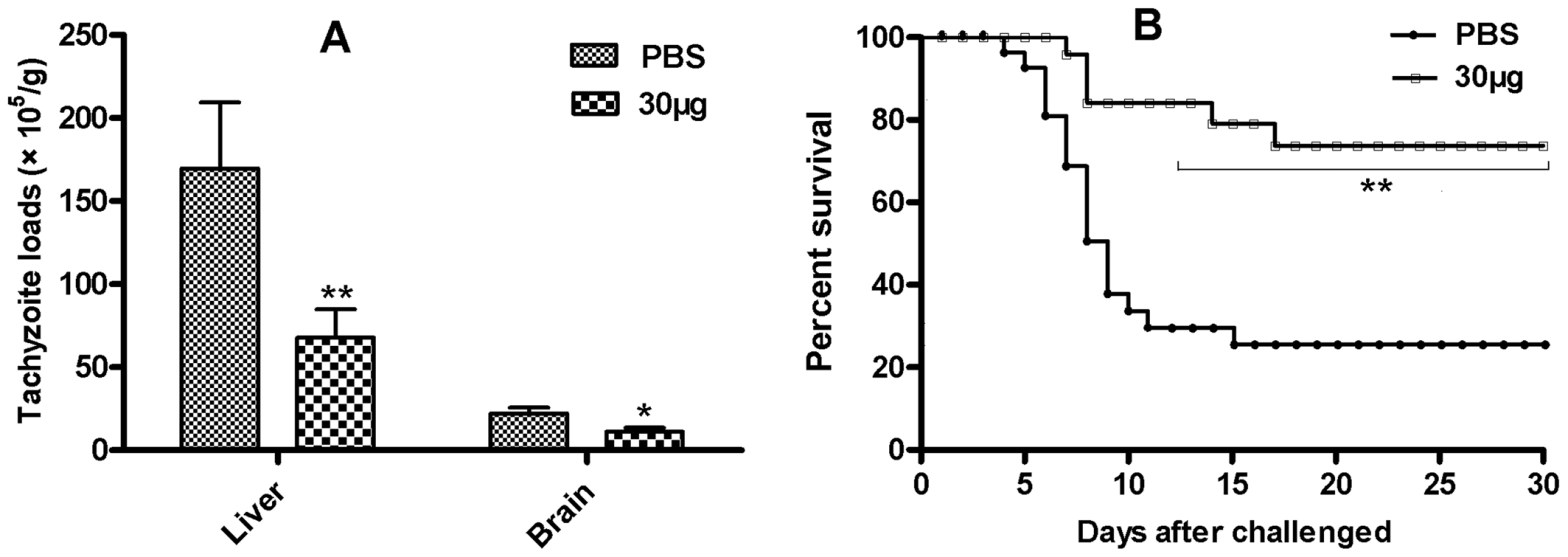
Assay for protection against oral challenge. Mice were nasally immunised with rTgACT or PBS. Two weeks after the last immunisation, mice were orally challenged with tachyzoites. (A) Mice from two groups (n = 8 PBS and 8 rTgACT) were orally infected with 1×10^4^ tachyzoites of the *T. gondii* RH strain. Liver and brain tachyzoite burdens were evaluated one month after challenge. (B) Mouse survival rates of the two groups (n = 12 PBS and 12 rTgACT) were monitored daily after challenge with 4×10^4^ tachyzoites of *T. gondii* RH strain until day 30 post-challenge. Differences in survival were significant (*P*<0.01). These results are representative of two independent experiments. Values are means ± SD. * *P*<0.05, ** *P*<0.01. PBS, phosphate buffered saline.

To evaluate the efficacy of the rTgACT antigen against *T. gondii* infection, the numbers of liver and brain tachyzoites in the mice were evaluated one month post-peroral challenge (1×10^4^ tachyzoites of RH strain). The tachyzoite loads of the mice given 30 µg of rTgACT were significantly reduced compared to the mice receiving PBS, showing 60.05% (*P*<0.01) and 49.75% (*P*<0.05) fewer tachyzoites in the liver and brain, respectively (Fig. 3A).

The tachyzoite loads of liver in mice were further estimated using qRT-PCR method. The results shown that the numbers of tachyzoites in 30 µg rTgACT group and control group were (6.93±1.71)×10^6^/g and (17.04±2.82)×10^6^/g, respectively. The tachyzoite reduction rate was 59.33%, which was in line with the results above described.

The survival rates of the mice were recorded daily following oral challenge (4×10^4^ tachyzoites of RH strain) until 30 days post-challenge. A significant increase in the survival rate was observed in the 30 µg rTgACT immunised group compared to the control group (*P*<0.05), indicating that rTgACT could induce partial protection against lethal challenge with the virulent RH strain of *T. gondii*. Fig. 3B shows the survival rates of the two groups. The mice immunised with 30 µg of rTgACT had a significantly increased survival rate (75%) compared to the PBS group (25%) on the 30th day after challenge. The survival rate was increased by 50% in the immunised mice compared to the control group (*P*<0.01). Death of the control mice immunised with PBS mostly occurred between days 6 and 9, and the mice immunised with rTgACT died between days 7 and 17 post-challenge. These results demonstrated a partial protective effect of rTgACT against both chronic and lethal *T. gondii* challenges.

The results demonstrated that immunisation with rTgACT not only reduced the tachyzoite burdens in the liver and brain of mice during chronic infection but also efficiently protected mice against a lethal challenge of *T. gondii*.

## Discussion

The vast majority of pathogens colonise and invade at mucosal surfaces. Preventing infections at these sites via mucosally active vaccines is a promising and rational approach for vaccine development. The stimulation of local immunity at the mucosal surfaces while also inducing systemic immunity has been performed for many years, although probably nowadays is considered more important [33]. As a mucosal pathogen, *Toxoplasma* infects humans and animals mainly through the digestive tract. Therefore, a developmental vaccine against *Toxoplasma* infection needs to induce protective responses at mucosal surfaces as a first line of defence. Additionally, because *Toxoplasma* has an intracellular phase in the blood and can disseminate throughout the host, it is important that the potential vaccine also induces systemic protective immune responses.

Most of the candidate vaccines have been given parenterally to stimulate systemic immunity, which is known to be very efficient in the control of toxoplasmosis [34]–[35]. Several studies have shown that nasal immunisation is an effective regimen to induce immune responses at mucosal effector sites, including the gut, genital tract, and nasal cavity [36]–[39].

Our group previously showed that nasal administration of *T. gondii* soluble tachyzoite antigen (STAg) in association with cholera toxin (CT) to BABL/c mice induced strong mucosal and systemic immune responses and conferred 85% survival against a lethal challenge [20]. The need for vaccines to have a controlled composition prompted us to repeat the mucosal vaccination trials with well-defined antigens. In the present study, we showed that nasal delivery of the purified recombinant *Toxoplasma gondii* actin (rTgACT) protein to BABL/c mice effectively reduced tachyzoite loads in the liver and brain and increased survival rates following oral challenge with the virulent RH strain. Systemic and mucosal antibody responses, as well as systemic cellular immunity to rTgACT, were mounted in these protected mice.

Previous studies confirmed that the recombinant proteins (such as GRA1) could increase immune responses and prolong animal survival against acute toxoplasmosis compared to DNA vaccinations [40]. In recent years, encouraging progress has been made in the identification of the recombinant protein vaccine candidates against *T. gondii* infection to extend survival time of mice [30]–[31], [40]–[45].

However, there is no fully available subunit vaccine against toxoplasmosis. In the present study, the protective effect of rTgACT against *T. gondii* infection in mice was evaluated. The vaccinated mice displayed significant protection against a lethal infection with the virulent RH strain (*P*<0.01 in survival rate) and against a chronic infection with the RH strain (60.05% and 49.75% reduction in brain and liver parasite loads, respectively) compared to the PBS-vaccinated control group. However, no relevant experimental reports had attempted to vaccinate mice with the recombination protein via intranasal route and then challenge orally with virulent *T. gondii* tachyzoites to evaluate protective immunity of a recombination protein.

The results of this experiment showed that immunising with rTgACT induced a high level of humoral antibodies and a mixed IgG1/IgG2a response with a predominance of IgG2a production. Increased production of not only Th1 (IFN-gamma and IL-2) but also Th2 (IL-4) and Treg (IL-10) was observed in the systemic immune response after vaccination in immunised mice, suggesting that Th1-, Th2- and Treg-type cells were generated. Compared to PBS, immunisation with rTgACT enhanced Th1-mediated immunity with high levels of IL-2 and IFN-γ, and as factors of Th2- and Treg-type immune response, a slight increase of IL-4 and IL-10 were observed. Therefore, these results demonstrated that rTgACT could elicit strong humoral and cellular Th1 immune responses, which are essential for cell-mediated immunity and resistance against intracellular pathogens. In conclusion, rTgACT elicits a strong specific Th1 immune response, partial Th2- and Treg-type immune responses, providing partial protection against both acute and chronic *T. gondii* infections.

IL-10 plays a key role in the regulation of many functions of the immune system [46]. This cytokine inhibits the proliferation of B and T lymphocytes and induces homeostasis in immune system responses [46]. Existing research results showed that the changes of IL-10 level in the supernatants of antigen-stimulated spleen cell cultures from mice immunised were different. Some studies which have other types of the vaccination, including DNA vaccine [47]–[50] and purified protein [51], against *T. gondii* have demonstrated increases in IL-10 level, but some findings which also using DNA vaccine [52], [53] and purified protein [30]–[31], [52] were not showed the change of IL-10 level. Recently, Abdollahi et al. showed that although serum levels of IL-10 were not changed at the early phases, they were elevated at the end phases (14 and 28 days) of vaccination with *T. gondii* E/SA [54]. The levels of IL-10 in mice immunised with rTgACT were increased 2 weeks after immunization in present study, which was in line with the result of Abdollahi et al. [54]. However, above studies have not to discuss why the changes of IL-10 appeared or not, so this is a interesting question requiring further discussion.

In light of the natural penetration of the intestinal tract by *T. gondii*, induction of a first line of local defence would be of great interest. Indeed, a great deal of evidence indicates that specific SIgA play a protective role against many pathogens that colonise mucosal tissues or invade the host organism by crossing mucous membranes [55]–[58]. Our previous study confirmed that intranasal immunisation with soluble tachyzoite antigen (STAg) in mice orally infected with *T. gondii* could increase the number of IgA secreting cells in the jejunum and ileum and could enhance immune barrier function of the small intestine of mice [59].

Our results showed that antigen-specific SIgA antibody titres in nasal, vaginal and intestinal washes were highest in the mice immunised nasally with greater than 20 µg rTgACT. These findings demonstrated that strong SIgA immune responses in mucosal sites were induced by nasal immunisation with rTgACT for the generation of protective immunity against *Toxoplasma*.

SIgA serves as a first line of defence in protecting the intestinal epithelium from enteric toxins and pathogenic microorganisms [15]. Indeed, *T. gondii* naturally invades the intestine of its host and can be partially controlled by nasal immunisation with *T. gondii* surface antigen 1 (SAG1) plus cholera toxin [60]. Our present work has demonstrated that this route of immunisation induces mucosal cells to participate in protective immunity. Thus, mucosal immunisation, particularly via the nasal route, has considerable potential for triggering immunity in all the mucosal and systemic compartments.

In summary, our study suggests that intranasal immunisation with rTgACT is a promising method to induce both systemic and local immune responses. Thus, it provides protection against infection by pathogenic organisms. According to previous studies and findings of this study, we assume that the protective immunity may be attributed to following factors, first of all, the strong systemic immune responses (including serum antibody titres in vivo, splenocyte stimulation index and production of cytokines in vitro) of the mice immunised with rTgACT may inhibit activity of tachyzoite in tissue and their proliferation in cells. Second, the high production of SIgA in mucosal washes from immunised mice, especially large number SIgA of intestinal mucosa may constitute a barrier in the small intestine mucosal surface to prevent the invasion of *T.gondii* tachyzoite, also may be the important cause of reducing tachyzoite loads in liver and brain.
